# Long-term survival of early-stage non–small cell lung cancer patients who underwent robotic procedure: a propensity score-matched study

**DOI:** 10.1186/s40880-016-0117-z

**Published:** 2016-07-07

**Authors:** Hao-Xian Yang

**Affiliations:** Department of Thoracic Surgery, State Key Laboratory of Oncology in South China, Collaborative Innovation Center for Cancer Medicine, Sun Yat-sen University Cancer Center, Guangzhou, 510060 Guangdong P. R. China

**Keywords:** Non–small cell lung cancer, Robot-assisted surgery, Long-term survival, Registry data

## Abstract

**Background:**

In the past decade, many researchers focused on to robot-assisted surgery. However, on long-term outcomes for patients with early-stage non–small cell lung cancer (NSCLC), whether the robotic procedure is superior to video-assisted thoracic surgery (VATS) and thoracotomy is unclear. Nonetheless, in the article titled “Long-term survival based on the surgical approach to lobectomy for clinical stage I non–small cell lung cancer: comparison of robotic, video assisted thoracic surgery, and thoracotomy lobectomy” by Yang et al. that was recently published in *Annals of Surgery*, the authors provided convincing evidence that the robotic procedure results in similar long-term survival as compared with VATS and thoracotomy. Minimally invasive procedures typically result in shorter lengths of hospital stay, and the robotic procedure in particular results in superior lymph node assessment.

**Main body:**

Our propensity score-matched study generated high-quality data. Based on our findings, we see promise in expanding patient access to robotic lung resections. In this study, propensity score matching minimized the bias involved between groups. Nevertheless, due to its retrospective nature, bias may still exist. Currently, the concept of rapid rehabilitation is widely accepted, and it is very difficult to set up a randomized controlled trial to compare robotic, VATS, and thoracotomy procedures for the treatment of NSCLC. Therefore, to overcome this limitation and to minimize bias, the best approach is to use a registry and prospectively collected, propensity score-matched data.

**Conclusions:**

Robotic lung resections result in similar long-term survival as compared with VATS and thoracotomy. Robot-assisted and VATS procedures are associated with short lengths of hospital stay, and the robotic procedure in particular results in superior lymph node assessment. Considering the alarming increase in the incidence of lung cancer in China, a nationwide database of prospectively collected data available for clinical research would be especially important.

## Background

Lung cancer is the most common malignancy worldwide and in China, with surgery being the most effective treatment for patients with early-stage disease [1, 2]. In the past decade, many researchers focused on robot-assisted surgery. In China, in 2015, there were only approximately 30 robotic surgical devices; however, just 1 year later, in 2016, there are over 50. Although robot-assisted surgery in China is in its infancy, the rapid increase in the number of robotic devices suggests that this procedure is becoming more and more popular. Considering that modern medical care values the concept of rapid rehabilitation of patients, it is not surprising that minimally invasive procedures, such as robot-assisted surgery, are drawing the interest of surgeons.

Currently, video-assisted thoracic surgery (VATS) is considered the most representative minimally invasive lobectomy procedure for the treatment of non–small cell lung cancer (NSCLC). However, VATS does have its disadvantages, such as an only two-dimensional view, camera tremor, and less freedom of instrumentation, resulting in a steep learning curve that hinders its widespread use [3]. Previous data suggested that although the percentage of VATS lung resections has been increasing over the past decade, in the 2010s in the United States, for example, thoracotomy was used in more than half of cases [4]; in China, the percentage of cases in which open thoracotomy was used was even larger. Owing to the advantages of three-dimensional optics, stable camera platform, and flexible instrumentation, the robotic procedure has been increasingly used in surgical resection of NSCLC. However, most previous studies of robotic lobectomy in the treatment of NSCLC focused on short-term outcomes, such as technique feasibility, surgical complications, and costs [5–8]; furthermore, long-term data are limited [9, 10], and survival comparisons between the robotic procedure and other more commonly used procedures, such as VATS and open procedures, are still lacking. Therefore, evaluating the long-term outcomes of patients with early-stage NSCLC who undergo robotic surgery, especially when compared with VATS and open procedures, is of considerable clinical interest.

## Main text

In the recent *Annals of Surgery* article entitled “Long-term survival based on the surgical approach to lobectomy for clinical stage I non–small cell lung cancer: comparison of robotic, video assisted thoracic surgery, and thoracotomy lobectomy” by Yang et al. [11], we compared the outcomes of patients with clinical stage I NSCLC who underwent robotic, VATS, and open lobectomy procedures. The purpose of our study was to evaluate the survival prognosis and operative morbidities of robotic lobectomy compared with the other two procedures [11]. We found that the 5-year overall survival (OS) rate for the robotic, VATS, and open lobectomy groups were 77.6%, 73.5%, and 77.9%, respectively; the differences were not statistically significant. The corresponding 5-year disease-free survival (DFS) rates were 72.7%, 65.5%, and 69.0%, respectively; here, the difference between the robotic and VATS groups was statistically significant (*P* = 0.047) [11]. However, multivariate analysis showed that surgical procedure was not an independent factor affecting OS and DFS [11]. Operative morbidities, except for survival outcomes, were also compared. Patients who underwent robotic lobectomy had similar lengths of hospital stay (median, 4 days) compared with those who underwent VATS but shorter lengths of hospital stay than those who underwent an open procedure (median, 5 days; *P* < 0.001) [11]. Interestingly, the robotic procedure harvested more stations of lymph nodes than VATS and open procedures (5 vs. 3 vs. 4; *P* < 0.001) [11]. Based on these findings, we see promise in expanding patient access to robotic lung resections. Notably, in our study, we used propensity score matching, thereby minimizing the bias involved between groups.

Nevertheless, the retrospective nature of our study is a limitation. Considering that the preferred surgical procedure varies dramatically among surgeons operating on patients with NSCLC, it is very difficult to conduct a randomized controlled clinical trial (RCT) to compare the long-term outcomes of patients undergoing robotic, VATS, and open surgical procedures. Currently, the concept of rapid rehabilitation is widely accepted. Furthermore, randomizing patients into undergoing either open thoracotomy or minimally invasive procedures may even present serious ethical concerns. Therefore, to overcome this limitation and to minimize bias, the best approach is to use a registry and prospectively collected, propensity score-matched data.

In China, a nationwide, unified prospective registry of complete clinical NSCLC data would be especially valuable. Unfortunately, such a registry does not currently exist. With increasing incidence and mortality, cancer is the leading cause of death in China [12]. Indeed, it was predicted that approximately 4,292,000 new cases of invasive cancer were diagnosed in 2015 [12]. In China, lung cancer is the most common cancer, accounting for approximately 17% of all cancers and constituting a major public health problem [12]. In today’s medical climate, the robot-assisted procedure for operable NSCLC faces two main challenges. First, it is still limited and used only in large-volume centers. Second, the cost of robotic technology, especially in a time of increasing healthcare expenses, may be a significant barrier. Nevertheless, we think that with advances in the robotic technique, device-related costs will decrease. Even so, we need reliable outcome data on which surgical procedure is best for operable NSCLC. Reliable data can be drawn only from reliable databases, and completeness is the most important factor in achieving a high-quality database. A nationwide, prospectively collected NSCLC database would provide data of the utmost value to clinical researchers. Future studies that rely on such a database would benefit from large case numbers, complete information, minimal bias, good representativeness, and generalizability.

## Conclusions

The study by Yang and colleagues [11] provided high-quality data suggesting that the robotic lobectomy procedure for the treatment of clinical stage I NSCLC results in similar long-term survival as compared with VATS and thoracotomy. Robot-assisted and VATS procedures are associated with shorter lengths of hospital stay, and the robotic procedure in particular results in superior lymph node assessment. Further studies comparing the benefits and indications of the robotic procedure are warranted. To build on this work, we strongly recommend the development of a high-quality, nationwide database with prospectively collected clinicopathologic information. Considering the alarming rise in the incidence of lung cancer in China, this database would be especially important. It would increase the number of cases that clinical studies could consider, minimize bias, and would therefore make the data more reliable.
